# Implementing Integrated Early Childhood Mental Health Services in Primary Care: Relationships, Vision, and Sustainability

**DOI:** 10.1007/s10488-023-01275-w

**Published:** 2023-06-05

**Authors:** Sameera S. Nayak, Arielle A. J. Scoglio, Shurobhi Nandi, Kayla Anderson, Daphney Mirand, Kate Roper, Larisa Méndez-Peñate, Christy Moulin, Malika Arty, Beth E. Molnar

**Affiliations:** 1 Department of Sociology, Anthropology, and Public Health, University of Maryland Baltimore County, Baltimore, MD USA; 2 Department of Applied and Natural Sciences, Bentley University, Waltham, MA USA; 3 University of Massachusetts Chan Medical School, Worcester, MA USA; 4 Institute for Health Equity & Social Justice Research, Northeastern University, 360 Huntington Avenue, Mail Stop INV 314, Boston, MA 02215 USA; 5 Massachusetts Department of Public Health, Bureau of Family Health and Nutrition, Boston, MA USA; 6 Early Childhood and Family Mental Health Program, Boston Public Health Commission, Boston, MA USA

**Keywords:** Early childhood mental health, Implementation, System of care, Family partner, Primary care, Integrated behavioral health

## Abstract

The Massachusetts Multi-City Young Children’s System of Care Project was a federally funded program to provide integrated early childhood mental health (ECMH) services in primary care for families of very young children (birth-six years old) with Serious Emotional Disturbances across three cities in Massachusetts, U.S.A. This study describes lessons learned from the implementation of this program and makes recommendations for best practices to improve the delivery and efficacy of ECMH services in primary care settings. Staff and leadership (n = 35) from 11 agencies (primary care practices, community service agencies, and local health departments) that co-implemented this program participated in focus groups and semi-structured key informant interviews. Thematic analysis was used to characterize specific facilitators and barriers to successfully implementing system-wide programming for ECMH. Four main themes were identified: (1) Strong multilevel working relationships are critical for integration, (2) Capacity-building activities can be leveraged to improve implementation, (3) Financial challenges are a primary barrier to building efficacious systems of care, and (4) Flexibility and resourcefulness can help overcome logistical challenges in integration. Implementation lessons learned may serve as guidance for other states and institutions in the U.S. seeking to improve the integration of ECMH services into primary care. They may also provide strategies to adapt and scale these interventions to improve the mental health and well-being of young children and their families.

## Introduction

Three out of five children in the United States experience at least one adverse childhood experience associated with health and behavioral difficulties across the life course (Gilbert et al., 2015; Kerker et al., 2015; Merrick et al., 2018). The prevalence of behavioral health challenges in children under six years is between 9–15% (Brauner & Stephens, 2006; Egger & Angold, 2006). Although behavioral intervention efforts for young children tend to focus on school-aged children, infants and children under six often need mental health support and services. Early experiences can have lasting health impacts, thus early intervention in this population is crucial (Bagner et al., 2014; Ramey & Ramey, 1998; Shonkoff & Garner, 2012). Utilizing a system of care model for improving children’s mental health can shift the focus from just the individual child to interventions that impact families and childhood environments (Hernandez & Hodges, 2003; Hodges et al., 2010).

Systems of care originated in efforts to improve services for children with mental health disorders and severe functional impairments (Stroul et al., 2002). The goal of a system of care is to create a network of multifaceted, multilevel interventions that bring agencies together in a coordinated manner to provide effective wraparound services (Cook & Kilmer, 2010; Hernandez & Hodges, 2003; Stroul et al., 2002). Implementation is challenging as agencies that provide mental health services for young children are often siloed and experience high staff turnover (Behar & Hydaker, 2009; Beidas et al., 2016; Hernandez & Hodges, 2003; Hodges et al., 2010; Jain et al., 2020).

Understanding facilitators and barriers to implementation is critical to improving interventions and adapting them to scale. This is especially important when developing multi-agency early childhood mental health (ECMH) programs because successfully addressing issues in early childhood requires bringing child-focused and family-level services together using a family-centered approach (Cook & Kilmer, 2010; Donney et al., 2022; Jain et al., 2020; Reichow et al., 2016).

The Massachusetts Multi-City Young Children’s System of Care Project (MA-SOC) was a Substance Abuse and Mental Health Services Administration (SAMHSA) funded primary care ECMH program implemented in three community service agencies in the three largest cities in Massachusetts, U.S.A. MA-SOC was an adapted replication of the evidence-based LAUNCH/MYCHILD model of integrated behavioral health for young children (www.ecmhmatters.org). This model has shown efficacy in improving caregiver mental health and child socio-emotional wellness over time (Molnar et al., 2018). It uses a family partner and clinician dyad to provide services and has successfully provided family-centered services that are well-received by families (Molnar et al., 2018; Nayak et al., 2021). It was also designated as a 2021 Best Practice by the Association of Maternal and Child Health Programs Innovation Station (www.amchp.org).

The aims of the current study were:

1. To explore how stakeholders in MA-SOC perceived facilitators of the project’s success and document challenges that they encountered.
2. To develop recommendations for improving the implementation of similar programs in other states.

Understanding barriers and facilitators of implementation can inform future statewide ECMH efforts. This can help ensure that efficacious initiatives are effectively executed and scaled-up. This study is novel in that it brings varied perspectives of stakeholders from multiple disciplines, including staff and leadership from the participating primary care practices, community service agencies, and local health departments across the state.

## Methods

### Overview

This was a qualitative study using data collected from semi-structured key informant interviews and focus groups. This study was approved by the Northeastern University Institutional Review Board.

### Program Description

MA-SOC created dedicated teams of family partners and intensive care coordinators to provide family-centered intensive care coordination ECMH services for young children (birth to six years old) with Serious Emotional Disturbances. Serious Emotional Disturbances are defined as “diagnosable mental, behavioral, or emotional disorder in the past year, which resulted in functional impairment that substantially interferes with or limits the child’s role or functioning in family, school, or community activities.” (SAMHSA, 2022).

The Children’s Behavioral Health Initiative is an interagency statewide initiative in Massachusetts to build a system of care for children and youth under the age of 21 with behavioral health needs (MDPH, 2022). Intensive care coordination is a service provided under this initiative. Intensive care coordinators work closely with children and families with behavioral health needs to achieve family-identified goals using individualized care plans and by coordinating multiple services. Family partners are peer-professionals with lived experience navigating systems to support the mental health of their own young children. They provide resources, systems navigation, and support to families of children with ECMH needs. Family partners could be actively supporting their own young children or have lived experience from supporting their own children in the past. Family partners have worked successfully in partnership with mental health clinicians in ECMH programs and group therapies. They can help build more meaningful relationships with the families they work with and can help increase parental participation in programs (Burton et al., 2014; Gopalan et al., 2015; Nayak et al., 2021).

The pediatric primary care practices that were part of MA-SOC identified families who might benefit from the program and referred them to these service delivery teams (intensive care coordinators and family partners). The service delivery teams were employed by community service agencies. These teams worked closely with families to provide bilingual and culturally sensitive services. In addition to primary care practices and community service agencies, city health departments played a crucial role in the program. These health departments facilitated the relationships and partnerships between the primary care agencies and the community service agencies involved with the project. In addition to direct service delivery, the MA-SOC program also conducted extensive training and workforce development activities to improve the ECMH capacity of the program teams as well as ECMH capacity statewide.

### Participants and Procedures

The study collected qualitative data through 3 focus groups and 19 semi-structured interviews with stakeholders in MA-SOC.

#### Focus Groups

Direct service staff (family partners and intensive care coordinators) were invited to participate in the focus groups through email invitations. MA-SOC was a four-year program. Focus groups were conducted in Year 1, Year 3, and Year 4 of the program.

#### Key Informant Interviews

We used a purposive sampling strategy to ensure adequate representation of leadership staff in semi-structured interviews. Members of the MA-SOC management team identified key leaders at each agency as important stakeholders who would have close knowledge of the program and insights into its implementation. Key leadership staff from the local health departments, the partnering community service agencies, and the partnering primary care site in each of the three cities were invited to participate in semi-structured interviews. Interviews were conducted in Year 2 (n = 12) and Year 4 (n = 7) of the program. Participants from the same agency could participate collaboratively in joint interviews. In Year 4 of the program, one primary care site did not respond to requests for interviews, and one was excluded as they had only recently joined the program.

As the focus groups and interviews were carried out at multiple time points during the program, some of the same participants were present in multiple interviews and focus groups. Two cities had 12 participants each, and one city had 11 participants, which resulted in a total of 35 unique participants: 8 intensive care coordinators, 6 family partners, 4 representatives from primary care practices, 11 representatives from community service agencies, and 6 representatives from local health departments.

#### Question Guides

In conjunction with the MA-SOC management team, the evaluation team developed interview guides for the focus groups and key informant interviews. The guides included various questions about participants’ perspectives on essential factors needed for replication, the status of behavioral health integration, and perspectives on the sustainability and financing of such projects. Questions remained consistent across years, across focus groups and interviews, and were conducted in English with members of the research team. We obtained informed consent verbally, and no personally identifying information was collected.

### Data Analysis

All interviews and focus groups were audio-recorded and transcribed. These data were analyzed collectively at the end of the study period. Codebook thematic analysis was used to analyze the data, identify cross-cutting ideas, and to understand shared experiences across participants (Braun & Clarke, 2006, 2014, 2019). Each focus group and interview transcript was treated as a separate data set. A multi-phase inductive approach was used to analyze the data (Braun & Clarke, 2006, 2014, 2019). First, two trained coders independently familiarized themselves with the data by reading the transcripts. Coders independently generated a set of initial data-driven codes. At this stage, these codes were compared, and a shared codebook was created. Coders used group discussion to collate codes into potential cross-cutting themes. In keeping with Braun & Clarke’s recommendations (2019), these discussions were not used to reach consensus but to share interpretations, co-develop perspectives, and form more nuanced ideas. Further group discussion with the larger research team was used to review themes, enhance confirmability, and develop clear names and definitions for each of the final themes identified. Members of the MA-SOC implementation team reviewed the final four themes to ensure the credibility of the results and interpretation. All analyses were conducted using NVivo 12 qualitative data analysis software (QSR International, 2020).

## Results

We identified four interrelated themes regarding the facilitators and barriers that participants perceived to be necessary for the successful implementation of a multi-agency ECMH system of care model: (1) Strong multilevel working relationships are critical for successful integration, (2) Capacity-building activities can be leveraged to build solidarity and improve implementation, (3) Financial challenges are a primary barrier to building efficacious systems of care, and (4) Flexibility and resourcefulness can help overcome logistical challenges in integration. Each theme is described below. Excerpts from participant quotes are embedded in the text using quotations; full example quotations are provided in Table 1.

**Table 1:** Summary of four main themes with example quotations

No	Theme	Example quotes^a^
1	Strong multilevel working relationships are critical for successful integration	“Being part of this grant, you have one partner, you’re learning and you get to finesse your craft. So, going into a home visit, finishing each other’s sentences and completing thoughts, the caregivers are so impressed by that, that they see us working well, then they have more trust in us and understanding to say that okay, this is going to work.” -Family Partner
		“We’re creating really nice systems for communication between the teams so that even if everyone had full staff turnover tomorrow, those systems could still survive- it's not about one person having one person’s email at an organization. So, I think as we are investing more and more internally to integrate our services… this has been a really nice way to pilot, at a pretty manageable level, between our organization and [community service agencies] that we have a long-standing great relationship with, and certainly had an opportunity to do more tight coordination and communication with.” -Primary Care Site Leader
2	Capacity-building activities can be leveraged to build solidarity and improve implementation	“Ongoing training, that promotes shared language, with primary care providers, providers and parents, regarding diagnosis and environment and needs and strengths and all that. That training, just bringing everybody together, with some shared language. It’s essential.” -Community Service Agency Leader
		“The grant is [a] multi-site grant and having it through [city A], [city B], and [city C] has been different and interesting in a way because you get to meet people from different cities and hear about their struggles and it’s good to know sometimes we have similar struggles as everybody else or if they’re doing something better, if they’re good at something-being able to learn from them has been great.” -Health Department Leader
3	Financial challenges are a primary barrier to building efficacious systems of care	“[T] here are benefits to [primary care sites] participating in the project, which I think they recognize, but they don’t have any staff time funded. And so, I think that can end up being a barrier, just in terms of the amount of time that they have to devote to the project. And I think if they did have some funded staff time, it would give them the ability to communicate with us a little bit more.” -Health Department Leader
		“We can’t live in this community anymore with the salaries that we pay. And as you heard, it’s super important that we have staff that are from the community, and understand the community, and then don’t turn over. We’re making relationships with families. Staff retention is a thing. It’s a problem, both at the health center and for us, and so I think if we could compensate our staff at comparative levels as hospitals and state agencies and school systems that would be great, because we’re as important in these families’ lives in terms of helping them, as the state agencies, hospitals, and schools.” -Community Service Agency Leader
4	Flexibility and resourcefulness can help overcome logistical challenges in integration	“I wanna say for the first time in a month, I talked to a parent in detail about what we did… how that was initiated was because the kiddo said “I wanna read a book,” and they have their own books in their [primary care center waiting] area, which doesn’t help either, and I kind of intervene and come over and say “I have this book—which one do you want?” And then, you know, parent came to give this back after the kiddo was done reading it, and that’s when I got in and interacted with them.” -Intensive Care Coordinator
		“We do have one of the clinical workers from [the community service agencies] going to [the health center] every single week to attend their huddles. So [community service agencies staff] goes every week to attend the huddles where they really meet to discuss different services, so having her there has been really helpful in terms of like putting a face to the work we’re doing and increasing awareness of the system of care model.” -Community Service Agency Leader

^a^Quotes are presented verbatim

### Theme 1. Strong Multilevel Working Relationships are Critical for Successful Integration

This theme captures the value of relationships in integration model implementation, both at the individual level between staff and at the systems level between agencies. First, direct service staff (family partners and intensive care coordinators) noted how their working relationship as a dyad had to be strong for them to provide high quality services. In traditional Children’s Behavioral Health Initiative models, family partners and intensive care coordinators do not necessarily work in exclusive dyads. Instead, intensive care coordinators might be paired with different family partners or other staff for different cases. In this study, stakeholders reported that working in MA-SOC as part of these exclusive dyads was preferable to traditional Children’s Behavioral Health Initiative and fostered stronger working relationships. Consistently working together as a dyad allowed the team to become more cohesive, “finesse” their craft, and gain greater trust. Participants reported that this exclusive dyad structure also allowed families to feel more confident in the services they received; they felt that this increased confidence in turn translated into more effective care for families.

Moreover, there was agreement across stakeholders that thinking about sustainability and relationship building needs to begin at the start of implementation and is necessary for successful integration. One way that was accomplished in this program was to choose community service agencies as the intervention sites. These agencies were already equipped to provide ECMH services and could potentially sustain the program after the grant funding ended. Participants noted that this approach can help set up systems and build agency-level relationships (e.g., workgroups and interagency collaborations) that last beyond funding from a single grant.

Participants perceived strong relationships across agencies as essential for the effective implementation of the model. They described the importance of identifying the right individuals within organizations to connect with. These point people could champion the model within their respective agencies and serve as a bridge between agencies. Shared values and well-aligned goals were described as extremely important when pursuing interagency collaborations. Participants highlighted the importance of developing relationships across agencies, particularly with those in positions of power who were able to make decisions regarding the program. By doing so, both agencies were able to maintain an investment in the partnership and keep the project moving even during times of staff turnover.

One positive outcome of building these relationships between agencies was better communication and shared patient management. Participants described “creating really nice systems for communication” that resulted in “more tight coordination and communication” and changed the working relationship between the community service agencies and the health centers. This led to in-person meetings and created a sense of teamwork and collaboration between staff from both agencies.

### Theme 2. Capacity-Building Activities Can Be Leveraged to Build Solidarity and Improve Implementation

In addition to direct service delivery, the MA-SOC program also held ECMH trainings, workshops, and learning sessions across the state. Some of these were only for MA-SOC staff; others were open to all providers and professionals interested in ECMH across the state. Attendees at these events came from a variety of disciplines including medical practitioners, community-based workers, and others working in ECMH in different sectors. Stakeholders identified these capacity-building activities as key elements they would like to see replicated in future ECMH programming. They felt these activities allowed them to share experiences across the different program sites. They explained that this was particularly important in the field of ECMH, where few providers have specialized training in mental health for very young children. Participants explained how these activities helped dismantle misconceptions and improved referrals and service delivery across systems.

Furthermore, participants felt that bringing together different types of providers for capacity-building activities helped infuse a “shared language” and vocabulary across sectors. This helped in “bringing everyone together” and building solidarity across agencies, fields, and cities. Participants also felt validated by the opportunity to share challenges and struggles with those from other agencies and cities. They reported how it boosted morale and sustained momentum. It also gave them opportunities to devise cross-discipline solutions together because of the unique perspectives different providers brought with them.

### Theme 3. Financial Challenges are a Primary Barrier to Building Efficacious Systems of Care

Barriers to the successful implementation described by stakeholders centered on financial challenges to building systems of care. For example, family partners and intensive care coordinators described a high level of administrative burden related to scheduling families, attending mandatory trainings and workshops, and entering required data into both electronic health records and grant-specific databases. They felt that these time and labor-intensive tasks compounded administrative stress and detracted from service delivery. Stakeholders reported that including funding for a designated point person at each site for these administrative tasks was essential to include in future scaled-up versions of the MA-SOC program. Similarly, they also deemed it essential to have a point person for behavioral health integration who oversaw and managed the model at the site level.

Difficulties with staff retention and inadequate compensation were also identified as significant challenges to implementation. Participants noted that turnover disrupts working relationships in the family partner and intensive care coordinator dyads. It also disrupts working relationships at the agency level if the main point of contact leaves. Participants explained that this disruption to relational continuity can impact progress at the family and agency levels. Inadequate compensation for intensive care coordinators, family partners, and other staff who work in community service agencies and community health centers contributed to this problem. Participants highlighted that staff compensation in community service agencies and community health centers were not comparable to those offered in other sectors (e.g., large hospital systems, school systems, and state agencies) and that increasing compensation could help in staff retention.

At the policy level, strategic recommendations suggested by stakeholders included (1) working to change insurance systems to allow billing for prevention work with very young children and (2) increasing overall rates of compensation for family partners and intensive care coordinators. In particular, highlighting the value of pay equity between intensive care coordinators and family partners was seen as transformative and necessary to increase respect for the family partner role. These recommendations to respect the role of the family partner and ensure that they receive parity in compensation are in line with efforts to formalize similar professions, such as community health workers. These efforts include having their work and expertise be recognized by insurers and the field (Gilkey et al., 2011).

Primary care sites also faced a challenge in dedicating staff time to the project without receiving grant funds to cover the expense. Staff at the community service agencies received grant funding for their positions; however, primary care sites were not reimbursed for staff participating in MA-SOC. Instead, primary care sites received funds to offset quality improvement costs to integrate behavioral health services. Without financial compensation, staff at the partnering primary care sites were not able to devote as much time to the program. Strategically selecting the right people within agencies with shared values and goals (as described in theme 1) helped ensure that key individuals remained invested in the partnerships and collaboratively navigated challenges to allow the model to function smoothly.

### Theme 4. Flexibility and Resourcefulness Can Help Overcome Logistical Challenges in Integration

A key aim of MA-SOC was to increase behavioral health integration into primary care by having the community service agencies teams be physically and administratively linked with the primary care sites. Stakeholders in our sample reported challenges with integration due to a lack of physical space and bureaucratic barriers, such as an inability to share electronic health records across agencies. The inability to collaborate electronically on patient care and logistical difficulties in sharing physical spaces were significant barriers to integration.

Stakeholders used creative strategies to navigate around these barriers in order to achieve higher levels of integration. For example, they engaged families in the waiting area of primary care sites and attended meetings and “huddles” to ensure that agencies could put “a face to the work” and remain connected during implementation. Participants described being resourceful and flexible to ensure that lack of physical integration and shared access to health records did not impede project success. They noted that these approaches improved patient care, enhanced feelings of collaboration and mutual respect, increased referrals from primary care sites to the team, and positively impacted working relationships between all agencies involved.

## Discussion

In this study of an integrated primary care ECMH program implemented across three cities, we identified a variety of factors which may inform the implementation of similar models. Our findings have important implications for other states implementing and scaling up similar models of ECMH systems of care services.

Stakeholders in our study identified relationship-building across staff and agencies as an important facet of implementation. Building such relationships includes identifying shared norms and values and investing in common goals in the vision for the model. Although the importance of multilevel alignment and investment for positive implementation is documented in the literature (Aarons et al., 2014; Behar & Hydaker, 2009; Swain et al., 2010), our findings suggest that this is especially important when there are minimal financial incentives. In programs such as MA-SOC, where multiple agencies are attempting to create a coordinated system of care, a lack of flexible funding poses a significant challenge. However, agencies can overcome these resource deficits if they have strong champions who have a deep investment in the goals of the program and who have partnerships with each other. States should pay close attention to the organizational values and individual goals of staff and leadership when selecting agencies and sites for participation in such programs.

Participants in our sample identified resources and support for staff, turnover rates, staffing infrastructure, and other financial logistics as important issues that affected implementation. Funding, systematic support, and prioritization are necessary to sustain innovative programs in public mental health agencies (Bond et al., 2014). Staff turnover, in particular, was a common challenge brought up by participants in this study and is a commonly identified issue in the mental healthcare workforce (Hyde, 2013). In MA-SOC, efforts to promote a culture of ECMH and build networks between primary care practices and behavioral health services were hindered by changes in staff. Staff turnover increases costs, limits the effectiveness of the organization, and negatively impacts staff morale, which plays a key role in performance and productivity when working with families (Aarons et al., 2012). However, participants in our sample identified a few key areas for intervention that could reduce this issue in future projects. Based on our findings, we first recommend building more administrative support into direct service programs to decrease exhaustion and burnout. Additionally, increased compensation rates and pay equity between staff (family partners and intensive care coordinators) is indicated. There is growing evidence of the value of peer professionals such as family partners in ECMH service-delivery (Burton et al., 2014; Gilkey et al., 2011; Nayak et al., 2021). Ensuring fair compensation for family partners is a factor in the professionalization of the role. Our findings spotlight the need for equitable reimbursement policies through insurance companies to compensate family partners and other peer professional services.

Access to evidence-based treatments and specialized clinics is a common challenge for families with young children needing mental health supports. Primary care can serve as an important entry point for children to receive these services if integration is done well (Davis et al., 2013). In our study, participants emphasized that integration into primary care was particularly difficult because the current models of care were designed for siloed health services. Stakeholders faced issues such as a lack of physical space, hindering physical integration, as well as a lack of access to shared electronic health records. Although participants described creative and flexible strategies to work around these challenges, these findings underscore the need to develop coordinated systems during implementation. When planning to integrate behavioral health into primary care settings, the implementation must include efforts to overcome physical, virtual, and electronic barriers to integration. This is one area where the value of strong relationships and buy-in from all agencies could be particularly salient. From a larger systems standpoint, these results suggest that healthcare policies must focus on improving secure data sharing to foster better inter-agency collaboration.

Moreover, for a project like MA-SOC to be successful, primary care practices must conceptualize ECMH services as essential to health care and incorporate behavioral health providers into the primary care team. Participants in this study highlighted the value of capacity-building activities in helping to increase knowledge in this area and drive this shift statewide. The workforce development activities of this program allowed stakeholders to build their networks and nurture supportive partnerships that increased motivation and strengthened the overall system for ECMH in the state. Such activities have the added benefit of expanding professional networks, which provide knowledge, advice, and expertise (Bunger et al., 2016). Professional networks allow key stakeholders to share ideas with one another to begin linking knowledge to action, which plays a major role in sustainability (Henry & Vollan, 2014). For states that might be trying to improve ECMH through a similar primary care-based program, we recommend designing projects with a larger vision in mind, ensuring that features of the project can help increase the quality of services provided in addition to the quantity.

### Limitations

Although a purposive sampling strategy allowed the researchers to gain perspectives from all relevant stakeholders, it is possible that stakeholders who chose to participate might have had a favorable opinion of the model and its implementation, leading to potential selection bias in the findings. Furthermore, the unique context of Massachusetts as a state with a robust public health system that has engaged in concerted efforts to build ECMH systems may reduce the transferability of these results to dissimilar state contexts.

## Conclusions

This study identified several implementation facilitators. These include the need to be strategic when choosing agencies to participate, prioritizing shared values and buy-in, focusing on increased compensation to reduce turnover, and developing ECMH projects inclusive of infrastructure and sustainability efforts. These results lay the foundation for future research into ECMH statewide program implementation. In particular, future research could focus on identifying what characteristics can help increase buy-in towards ECMH programs and how best to use workforce development activities to reach a broader cadre of ECMH stakeholders within a given state. Testing and piloting strategies to increase the integration of behavioral health in primary care is also warranted. Results from this study can serve as a preliminary guide for states who are interested in improving ECMH through publicly funded programming and developing long-term sustainable systems of care for young children’s mental health and well-being.

## Declarations

### Methods Guidelines

Qualitative methods guidelines were used.

## References

[CR1] Aarons GA, Ehrhart MG, Farahnak LR, Sklar M (2014). Aligning leadership across systems and organizations to develop a strategic climate for evidence-based practice implementation. Annual Review of Public Health.

[CR2] Aarons GA, Fettes DL, Sommerfeld DH, Palinkas LA (2012). Mixed methods for implementation research: Application to evidence-based practice implementation and staff turnover in community-based organizations providing child welfare services. Child Maltreatment.

[CR3] Bagner DM, Frazier SL, Berkovits MD (2014). Getting ready for preschool: linking early intervention and family mental health for infants and toddlers with developmental delay. Administration and Policy in Mental Health.

[CR4] Behar L, Hydaker WM (2009). Defining community readiness for the implementation of a system of care. Administration and Policy in Mental Health.

[CR5] Beidas RS, Marcus SC, Wolk CB, Powell BJ, Aarons GA, Evans AC, Hurford MO, Hadley TR, Adams DR, Walsh LM, Babbar S, Barg FK, Mandell DS (2016). A prospective examination of clinician and supervisor turnover within the context of implementation of evidence-based practices in a publicly-funded mental health system. Administration and Policy in Mental Health.

[CR6] Bond GR, Drake RE, McHugo GJ, Peterson AE, Jones AM, Williams JR (2014). Long-term sustainability of evidence-based practices in community mental health agencies. Administration and Policy in Mental Health.

[CR7] Braun V, Clarke V (2006). Using thematic analysis in psychology. Qualitative Research in Psychology.

[CR8] Braun V, Clarke V (2014). What can “thematic analysis” offer health and wellbeing researchers?. International Journal of Qualitative Studies on Health and Well-Being.

[CR9] Braun V, Clarke V (2019). Reflecting on reflexive thematic analysis. Qualitative Research in Sport, Exercise and Health.

[CR10] Brauner CB, Stephens CB (2006). Estimating the prevalence of early childhood serious emotional/behavioral disorders: Challenges and recommendations. Public Health Reports.

[CR11] Bunger AC, Hanson RF, Doogan NJ, Powell BJ, Cao Y, Dunn J (2016). Can learning collaboratives support implementation by rewiring professional networks. Administration and Policy in Mental Health.

[CR12] Burton M, Cohen AK, Cohen AK, Jain-Aghi S (2014). Family partners improve early childhood mental health services. Psychiatric Services.

[CR13] Cook JR, Kilmer RP (2010). Defining the scope of systems of care: An ecological perspective. Evaluation and Program Planning.

[CR14] Davis MM, Balasubramanian BA, Waller E, Miller BF, Miller BF, Green LA, Cohen DJ (2013). Integrating behavioral and physical health care in the real world: Early lessons from advancing care together. Journal of the American Board of Family Medicine.

[CR15] Donney JF, Ghandour RM, Kogan MD, Lewin A (2022). Family-centered care and flourishing in early childhood. American Journal of Preventive Medicine.

[CR16] Egger HL, Angold A (2006). Common emotional and behavioral disorders in preschool children: Presentation, nosology, and epidemiology. Journal of Child Psychology and Psychiatry.

[CR17] Gilbert LK, Breiding MJ, Merrick MT, Thompson WW, Ford DC, Dhingra SS, Parks SE (2015). Childhood adversity and adult chronic disease: An update from ten states and the District of Columbia, 2010. American Journal of Preventive Medicine.

[CR18] Gilkey M, Garcia CC, Rush C (2011). Professionalization and the experience-based expert: Strengthening partnerships between health educators and community health workers. Health Promotion Practice.

[CR19] Gopalan G, Chacko A, Franco LM, Dean-Assael K, Rotko L, Marcus SM, Hoagwood K, McKay MM, McKay M (2015). Multiple family groups for children with disruptive behavior disorders: Child outcomes at 6-month follow-up. Journal of Child and Family Studies.

[CR20] Henry AD, Vollan B (2014). Networks and the challenge of sustainable development. Annual Review of Environment and Resources.

[CR21] Hernandez M, Hodges S (2003). Building upon the theory of change for systems of care. Journal of Emotional and Behavioral Disorders.

[CR22] Hodges S, Ferreira K, Israel N, Mazza J (2010). Systems of care, featherless bipeds, and the measure of all things. Evaluation and Program Planning.

[CR23] Hyde, P. (2013). *Report to congress on the Nation’s substance abuse and mental health workforce issues*. U.S. Dept. for Health and Human Services. Retrieved from https://www.openminds.com/wp-content/uploads/indres/012413samhsabhworkforce.pdf.

[CR24] Jain S, Jain S, Reno R, Cohen AK, Bassey H, Master M, Nichols CR (2020). A family-centered mixed-methods needs assessment for the system of care for young children with social-emotional and behavioral concerns. Children and Youth Services Review.

[CR25] Kerker BD, Zhang J, Nadeem E, Stein REK, Hurlburt MS, Heneghan AM, Heneghan A, Heneghan AM, Landsverk J, Horwitz SM (2015). Adverse childhood experiences and mental health, chronic medical conditions, and development in young children. Academic Pediatrics.

[CR26] MDPH. (2022). *Children’s Behavioral Health Initiative (CBHI) | Mass.gov*. https://www.mass.gov/childrens-behavioral-health-initiative-cbhi

[CR27] Merrick MT, Ford DC, Ports KA, Guinn AS (2018). Prevalence of adverse childhood experiences from the 2011–2014 behavioral risk factor surveillance system in 23 states. JAMA Pediatrics.

[CR28] Molnar BE, Lees KE, Roper K, Byars N, Méndez-Peñate L, Moulin C, McMullen W, Wolfe J, Allen D (2018). Enhancing early childhood mental health primary care services: Evaluation of MA Project LAUNCH. Maternal and Child Health Journal.

[CR29] Nayak SS, Tobias C, Wolfe J, Roper K, Méndez-Peñate L, Moulin C, Arty M, Scoglio AAJ, Kelleher A, Rue J, Brigham M, Bradshaw T, Byars N, Camacho A, Douglas S, Molnar BE (2021). Engaging and supporting young children and their families in early childhood mental health services: The role of the family partner. Community Mental Health Journal.

[CR30] QSR International. (2020). *NVivo (Version 12)*. https://www.qsrinternational.com/nvivo-qualitative-data-analysis-software/home

[CR31] Ramey CT, Ramey SL (1998). Early intervention and early experience. American Psychologist.

[CR32] Reichow B, Boyd BA, Barton EE, Odom SL (2016). Handbook of early childhood special education.

[CR33] SAMHSA. (2022, November 22). *Mental Health and Substance Use Disorders*. https://www.samhsa.gov/find-help/disorders

[CR34] Shonkoff JP, Garner AS (2012). The lifelong effects of early childhood adversity and toxic stress. Pediatrics.

[CR35] Stroul BA, Pires SA, Armstrong MI, Zaro S (2002). The impact of managed care on systems of care that serve children with serious emotional disturbances and their families. Children’s Services.

[CR36] Swain K, Whitley R, McHugo GJ, McHugo GJ, McHugo GJ, Drake RE (2010). The sustainability of evidence-based practices in routine mental health agencies. Community Mental Health Journal.

